# Thrombospondin-1 Is Necessary for the Development and Repair of Corneal Nerves

**DOI:** 10.3390/ijms19103191

**Published:** 2018-10-16

**Authors:** Yukako Tatematsu, Qalbi Khan, Tomas Blanco, Jeffrey A. Bair, Robin R. Hodges, Sharmila Masli, Darlene A. Dartt

**Affiliations:** 1 Schepens Eye Research Institute/Massachusetts Eye and Ear, Department of Ophthalmology, Harvard Medical School, Boston, MA 02114, USA; yuka.tatematsu@gmail.com (Y.T.); Qalbi.khan@uit.no (Q.K.); tomas_blanco@meei.harvard.edu (T.B.); Jeffrey_Bair@meei.harvard.edu (J.A.B.); robin_hodges@meei.harvard.edu (R.R.H.); 2 Department of Medical Biology, Faculty of Health Sciences, University of Tromsø, Tromsø 9037, Norway; 3 Department of Ophthalmology, Boston University School of Medicine, Boston, MA 02118, USA; smasli@bu.edu

**Keywords:** thrombospondin-1, dry eye, cornea, sensory nerves, CGRP, Substance P

## Abstract

Thrombospondin-1-deficient (TSP-1^−/−^) mice are used as an animal model of Sjögren’s Syndrome because they exhibit many of the symptoms associated with the autoimmune type of dry eye found in primary Sjögren’s Syndrome. This type of dry eye is linked to the inflammation of the lacrimal gland, conjunctiva, and cornea, and is thought to involve dysfunction of the complex neuronal reflex arc that mediates tear production in response to noxious stimuli on the ocular surface. This study characterizes the structural and functional changes to the corneal nerves that are the afferent arm of this arc in young and older TSP-1^−/−^ and wild type (WT) mice. The structure and subtype of nerves were characterized by immunohistochemistry, in vivo confocal microscopy, and confocal microscopy. Cytokine expression analysis was determined by Q-PCR and the number of monocytes was measured by immunohistochemistry. We found that only the pro-inflammatory cytokine MIP-2 increased in young corneas of TSP-1^−/−^ compared to WT mice, but tumor necrosis factor-α (TNF-α), monocyte chemoattractant protein-1 (MCP-1), and macrophage inflammatory protein-2 (MIP-2) all increased in older TSP-1^−/−^ mouse corneas. In contrast, CD11b+ pro-inflammatory monocytes did not increase even in older mouse corneas. Calcitonin gene-related peptide (CGRP)-, but not Substance P (SubP)-containing corneal nerves decreased in older, but not younger TSP-1^−/−^ compared to WT mouse corneas. We conclude that CGRP-containing corneal sensory nerves exhibit distinct structural deficiencies as disease progresses in TSP-1^−/−^ mice, suggesting that: (1) TSP-1 is needed for the development or repair of these nerves and (2) impaired afferent corneal nerve structure and hence function may contribute to ocular surface dysfunction that develops as TSP-1^−/−^ mice age.

## 1. Introduction

Dry eye disease is currently classified into two major categories: evaporative dry eye (EDE) and aqueous tear-deficient dry eye (ADDE) [1]. EDE is characterized by excessive water loss from the ocular surface despite normal lacrimal gland function, while the primary cause of ADDE is insufficient lacrimal secretion. The hyperosmolarity of the tear film observed in ADDE, a result of the reduction in lacrimal gland fluid secretion, inflames the underlying ocular surface. ADDE is further divided into two major subclasses based on etiological causes: Sjögren’s Syndrome Dry Eye (SSDE) and Non-Sjögren’s Syndrome Dry Eye (NSSDE).

Sjögren’s Syndrome (SS) is a prevalent autoimmune disorder [2], affecting approximately 3–4% of adults [1] and disproportionately affecting women at a ratio of approximately 9:1 [2]. This disease involves the chronic inflammation of selected exocrine glands, producing xerostomia (dry mouth) and xerophthalmia (dry eye). The manifestations of dry eye are linked to the inflammation of the cornea, conjunctiva, and lacrimal gland [1].

The cornea is one of the most densely innervated structures in the entire human body. Histological studies of the corneal epithelium reveal nerve bundles in the underlying stroma that penetrate the Bowman’s membrane and branch into smaller bundles subjacent to the epithelium. These nerves running parallel to the corneal surface are known as the subbasal plexus. From the subbasal nerves, smaller individual fibers penetrate into different epithelial cell layers, terminating close to the surface as free nerve endings [3]. The majority of these nerves are sensory and the nerve endings release neurotransmitters, mainly Substance P (SubP) and calcitonin gene-related peptide (CGRP), in response to noxious stimuli mediating the inflammatory response.

Irritants interacting with the ocular surface and other stimuli, such as temperature, trigger the complex neural reflex arc that stimulates the production of tears. This arc consists of afferent sensory corneal nerves, the trigeminal ganglion, higher level ganglia and brain areas, and efferent parasympathetic and sympathetic nerves that innervate the conjunctiva, lacrimal gland, and meibomian gland. Activation of the efferent nerves causes fluid production from these tissues that combine to form tears. Thus, loss or changes in the sensitivity of the afferent corneal nerves would decrease their activity, lower tear volume, and alter the composition of the tears. This change in tear production can cause dry eye [2,4]. Using confocal microscopy, Villani et al. reported that SS patients show a decrease in both the number and density of corneal nerve fibers in the subbasal plexus [5].

Thrombospondin-1 (TSP-1) is a matricellular protein that activates latent transforming growth factor (TGF)-β, a potent anti-inflammatory cytokine. TSP-1 interacts with cell surface receptors, growth factors, and extracellular matrix proteins to regulate extracellular and intracellular signaling complexes [6]. TSP-1 is expressed in the corneal epithelium, stroma, and endothelium. To demonstrate the role of TSP-1 in ocular surface function, we used the TSP-1-deficient (TSP-1^−/−^) mouse model. In both sexes this mouse appears to be normal at birth, but with age progressively develops dry eye and the aberrant histopathology associated with Sjögren’s Syndrome, making it a novel mouse model of dry eye [7]. In the lacrimal glands of TSP-1^−/−^ mice the peripheral nerves are injured, preventing the release of neurotransmitters and decreasing protein secretion, thus leading to dry eye [8]. In the malfunctioning lacrimal glands, an increase in apoptosis and deterioration is observed with the production of CD4+ T-rich inflammatory infiltrates, similar to those seen with Sjögren’s Syndrome. On the ocular surface, the lack of TSP-1 leads to the disruption of the corneal epithelial layer, corneal edema, and a reduction in conjunctival goblet cell density [7].

Around the age of 12 weeks, the presence of anti-Sjögren’s Syndrome antigen A and B antibodies were confirmed in the serum of TSP-1^−/−^ mice. A loss of function of the lacrimal glands and therefore the tear production was suggested to explain the changes observed in the cornea [7,9]. A decrease in corneal nerve function could contribute to the decreased tear film and the development of dry eye; however, the structure and function of corneal nerves, specifically the sensory nerves and nerve endings, in the TSP-1^−/−^ mouse has yet to be investigated.

The present study aims to determine if the structure of nerves, specifically neurotransmitter-containing nerves, and infiltrating cells was altered in the corneas of TSP-1^−/−^ compared to the control wild type mice. We performed histological, immunohistochemical, and in vivo confocal analyses to assess any changes in nerve structure and we observed a decrease in corneal nerve density in TSP-1^−/−^ mice compared to their WT counterparts in the control group. Cytokine analysis also revealed an increase in the expression of dry eye-associated inflammatory mediators in the cornea.

## 2. Results

### 2.1. With Hematoxylin and Eosin Staining, Corneal Epithelial and Stromal Morphology Was Similar in WT and TSP-1^−/−^ Mouse Corneas

**Figure 1:**
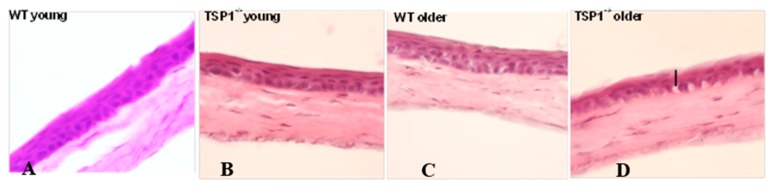
Hematoxylin and Eosin staining of corneas from young (4-week-old) wild type (**A**), young (4-week-old) thrombospondin-1-deficient (TSP-1^−/−^) (**B**), older (12-week-old) wild type (**C**), and older (12-week-old) TSP-1^−/−^ (**D**) mice showed no apparent inflammatory cells. No apparent morphological changes were observed between 4-week-old mice. Twelve-week-old TSP-1-deficient mice displayed a thinner epithelial layer and an uneven line of basal epithelial cells containing interspersed elongated cells with vacuoles (indicated with a black bar).

To assess morphological changes and inflammatory cell infiltration in the cornea, hematoxylin and eosin (H-E) staining was performed in young (4-week-old) and older (12-week-old) female TSP-1^−/−^ and control mice (Figure 1A–D). In older TSP-1^−/−^, but not WT mice, a thinner epithelial layer and an uneven line of basal epithelial cells containing interspersed elongated cells with vacuoles were observed in the cornea (Figure 1D). No apparent inflammatory cells such as neutrophils were observed in either strain.

### 2.2. When Analyzed by Real-Time PCR, MCP-1, MIP-2, and TNF-α Expression Was Increased in TSP-1^−/−^ Compared to WT Mouse Corneas

**Figure 2:**
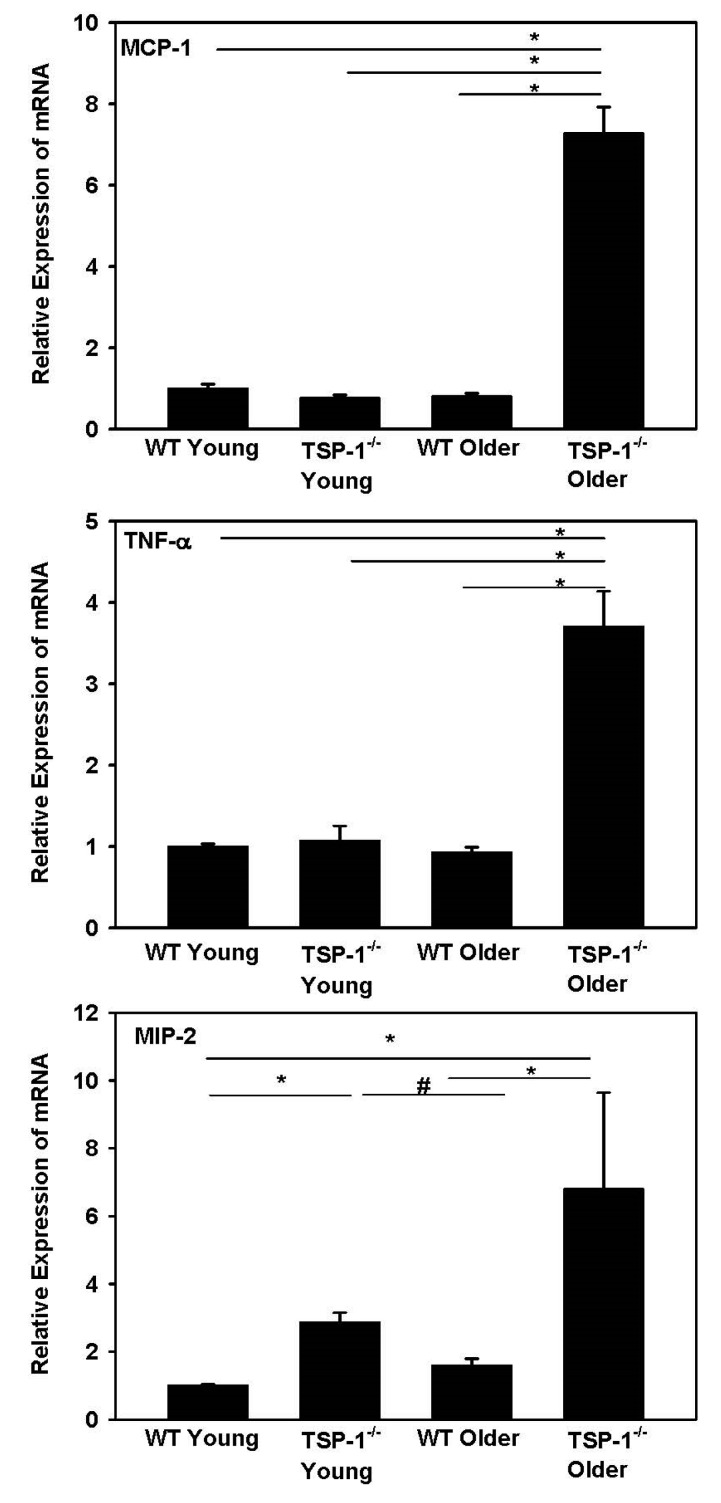
Comparison of monocyte chemoattractant protein-1 (MCP-1), macrophage inflammatory protein-2 (MIP-2), and tumor necrosis factor-α (TNF-α) expression in wild type (WT) and TSP-1^−/−^ mouse corneas by quantitative real-time PCR. *n* = 3. * and # denote significant differences.

To investigate the inflammation reactions at the molecular level, real-time PCR analysis of selected chemokines/cytokines was performed on mRNA harvested from corneal tissue. Monocyte chemoattractant protein-1 (MCP-1), tumor necrosis factor-α (TNF-α), and macrophage inflammatory protein-2 (MIP-2) expression was detected in all corneas tested. There was a significant increase in all three cytokines in older (12-week-old) TSP-1^−/−^ compared to age-matched WT corneas (Figure 2). The expression of MIP-2 in TSP-1^−/−^ mice was significantly increased compared to that in WT mice for both young and older mice (*p* < 0.05).

### 2.3. The Number of Monocytes Was Similar in WT and TSP-1^−/−^ Mouse Corneas

**Figure 3:**
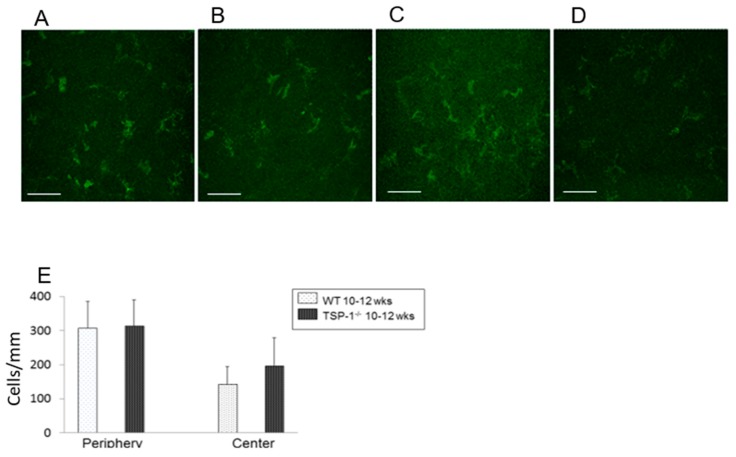
Comparison of the number of monocytes in WT and TSP-1^−/−^ mouse corneas through analysis of CD11b+ cells (**E**). CD11b+ cells were present in young WT peripheral areas (**A**), young WT central areas (**B**), young TSP-1^−/−^ peripheral areas (**C**), and young central areas (**D**). The number of cells/mm was analyzed for each condition and are presented in (**E**). *n* = 3. Scale bar represents 50 μm.

CD11b+, a marker for monocyte-derived cells, was utilized to quantify inflammatory cells by immunofluorescence microscopy in corneal whole mounts. Peripheral and central corneas were analyzed separately. CD11b+ cells were present in WT and TSP-1^−/−^ mouse corneas in both peripheral and central areas (Figure 3A–D). There was no statistically significant difference between the number of CD11b+ cells in WT and TSP-1^−/−^ mice in either peripheral or central cornea (Figure 3E). The densities of CD11b+ of the cornea in periphery were 307.712 ± 77.76 and 313.6 ± 75.90 cells/mm^2^ (*n* = 3) in WT and TSP-1^−/−^ mice, respectively. The density of cells in the central cornea was less than that in the periphery in both types of corneas with a density of 142.91 ± 52.10 cells/mm^2^ (*n* = 3) in the WT central cornea and 197.31 ± 81.736 cells/mm^2^ (*n* = 3) in the TSP-1^−/−^ central cornea (Figure 3E).

### 2.4. Number of Corneal Nerves Was Decreased in TSP-1^−/−^ Compared to WT Mice When Analyzed by In Vivo Confocal Microscopy

**Figure 4:**
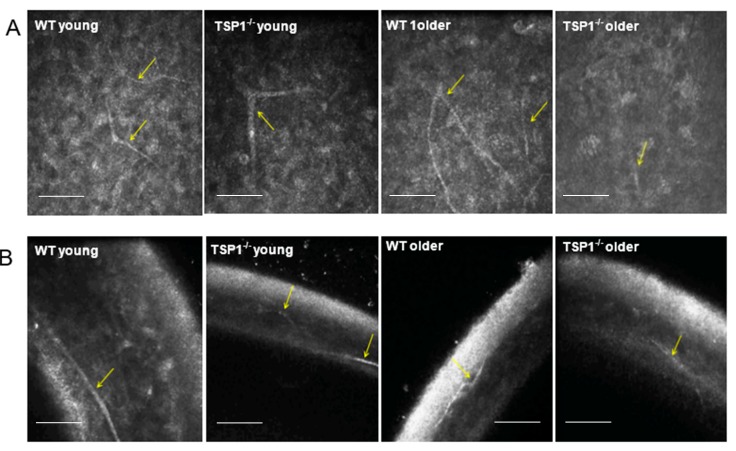
In vivo confocal images from the corneal epithelium of young (**A**) and older (**B**) mice. Branches of corneal nerves emerging from the nerve trunk are indicated by yellow arrows. Scale bars represent 100 μm. *n* = 3 for all conditions.

To assess changes in corneal nerve structure, in vivo confocal images from the corneal epithelium through the stroma were recorded (Supplemental data movies). Single representative micrographs are shown in Figure 4. Branches of corneal nerves emerging from the nerve trunk (Figure 4, yellow arrows) were visible both in oblique (Figure 4A) and transverse (Figure 4B) sections. No apparent difference was observed between the younger groups.

Fewer corneal nerves were observed in older (12-week-old) WT compared to those in younger (4-week-old) WT mice. Additionally, fewer corneal nerves were present older TSP-1^−/−^ compared to older WT mice (*n* = 3). Furthermore, the nerves present in the older TSP-1^−/−^ mouse corneas appeared to be discontinuous.

### 2.5. Corneal Nerve Density Was Decreased in TSP-1^−/−^ Compared to WT Mice When In Vitro Immunofluorescence Microscopy Analysis Was Used

**Figure 5:**
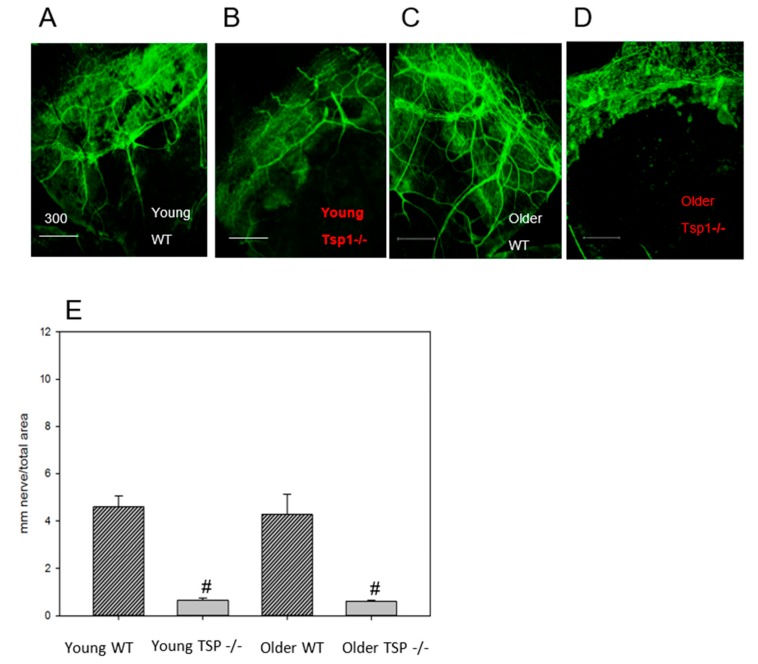
Whole corneas were fixed and stained with anti-Class III β-tubulin antibody. The number of nerves, number of nerve branches, and nerve density were measured and compared across groups. Both young (4-week-old) and older (12-week-old) WT mice displayed abundant nerve branches emerging from the nerve trunk near the limbus area (**A**,**C** respectively). The nerve density in corneas from TSP-1^−/−^ mice was significantly lower than that in age-matched WT counterparts (**B**,**D**,**E**). *n* = 6 for all groups. Scale bars represent 300 μm and # denotes significance from WT animals.

As an additional measure of corneal nerve density, whole corneas were fixed and stained with anti-Class III β-tubulin antibody. Nerve density was measured and compared across groups (Figure 5). Both young and older WT mice displayed abundant nerve branches emerging from the nerve trunk near the limbus area (Figure 5A,C). Young TSP-1^−/−^ corneas (Figure 5A,B) contained fewer branches as compared to their WT counterparts. Similarly, in older TSP-1^−/−^ mouse corneas, relatively few nerves were observed when compared to the older WT mice (Figure 2C,D). In addition, a decrease in both nerve diameter and number of branches was observed in older TSP-1^−/−^ mice when compared to the corresponding WT mouse corneas.

Corneal nerve density in the peripheral area was quantified by tracing nerves according to established methods based on the tubular nature of the staining with anti-Class III β-tubulin [10] and are reported as average mm nerve/mm^2^ ± SD (Figure 5E). Young WT corneal nerves had significantly increased density as compared to young TSP-1^−/−^ corneas (*n* = 6) and corneal nerve density in older WT was significantly greater than that in older TSP-1^−/−^ corneas.

### 2.6. CGRP-, but not SubP-Containing Corneas Nerves Accounted for the Change in Nerve Staining with Age and Disease

**Figure 6:**
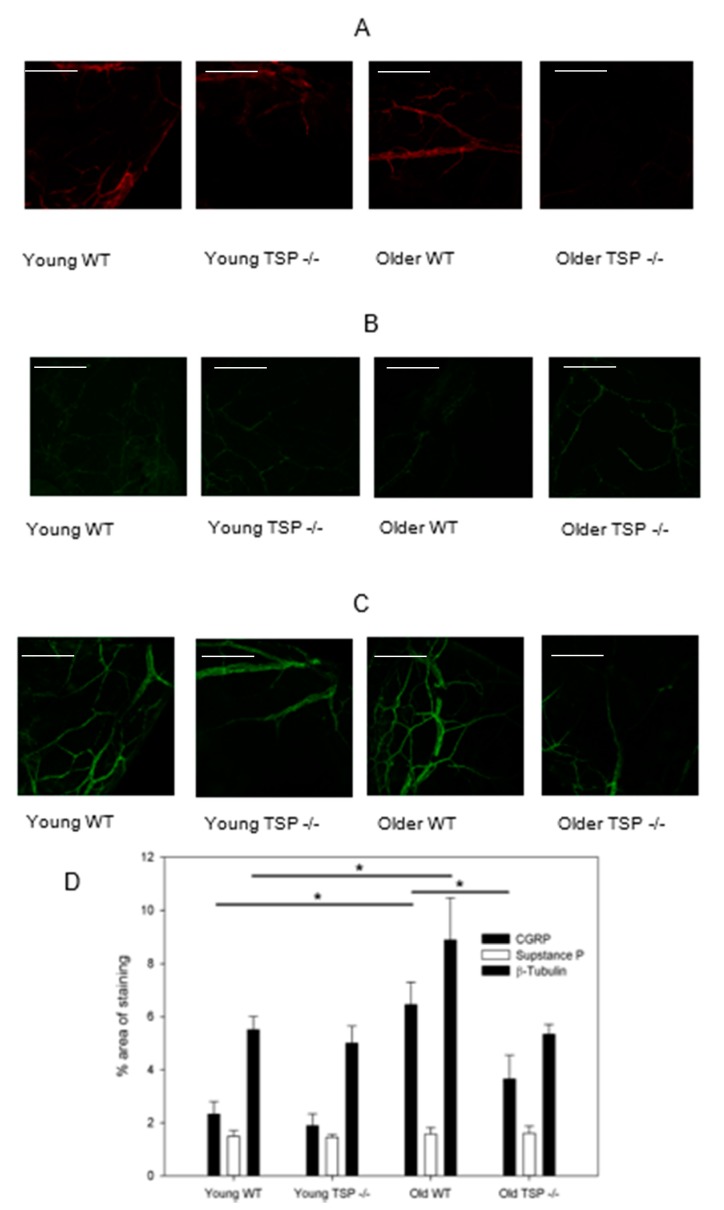
Examination of calcitonin gene-related peptide (CGRP)- (**A**) and Substance P (SubP)- (**B**) containing cells in WT and TSP-1^−/−^ mice by in vitro immunofluorescence microscopy analysis. The average percent area signaling for the protein of interest was measured using ImageJ (Fiji) due to the discrete nature of neurotransmitter-containing vesicles. The percent area of staining was also examined for β-tubulin staining (**C**) and compared across groups (**D**). *n* = 6 for all conditions. Scale bars represent 300 μm and * denotes a significant difference.

In order to assess nerve sub-types, we quantified the number of nerves containing two of the major neurotransmitters in sensory nerves, CGRP and SubP. To determine if the number of CGRP- and SubP-containing sensory nerves were different in WT compared to TSP-1^−/−^ mouse corneas, immunofluorescence analysis was performed using anti-SubP or anti-CGRP antibodies. All sections were double-labeled with anti-Class III β-tubulin antibody as a control. For this analysis, the average percentage of area stained for the specific neurotransmitter was measured using ImageJ (Fiji) due to the discrete nature of neurotransmitter-containing vesicles stained by anti-CGRP or anti-SubP antibodies. For anti-CGRP immunoreactivity, the percent area was unchanged between young WT and young TSP-1^−/−^ corneas, being 2.4 ± 0.47% and 1.9 ± 0.44%, respectively. For older mice the percent area of CGRP was significantly increased to 6.5 ± 0.85% for WT compared to 3.6 ± 0.89 for TSP-1^−/−^ corneas (Figure 6A,D). The percent area of CGRP staining in older WT mouse corneas was also higher compared to the results in young WT mouse corneas (*p* < 0.05).

The percent area of anti-SubP immunoreactivity was not different at either age or either strain of mouse cornea and was measured to be 1.49 ± 0.22% for young WT, 1.5 ± 0.10% for young TSP-1^−/−^, 1.6 ± 0.25% for older WT, and 1.6 ± 0.28% for older TSP-1^−/−^ corneas (Figure 6B).

For anti-Class III β-tubulin immunoreactivity, the percent area was measured to be 5.5 ± 0.51% for young WT, 5.0 ± 0.64% for young TSP-1^−/−^, 8.9 ± 1.6% for older WT, and 5.3 ± 0.38% for older TSP-1^−/−^ corneas. Younger WT corneas compared to older WT corneas demonstrated a significant decrease in the area of corneal nerves stained for anti-Class III β-tubulin comp (*p* < 0.05). Thus CGRP-, but not SubP-containing nerves accounted for the increase in the percent area of corneal nerves in older WT corneas and the decrease in older TSP-1^−/−^ compared to WT mouse corneas.

## 3. Discussion

Dry eye from lacrimal gland and ocular surface dysfunction is widely associated with inflammation. Especially in autoimmune dry eye, an upregulation of proinflammatory cytokines, chemokines, and metalloproteinases has been demonstrated [11]. The increase in these inflammatory factors leads to a rapid expansion of autoreactive T cells which then migrate to the conjunctiva, lacrimal gland, and cornea, causing inflammation and dysfunction [12]. In TSP-1^−/−^ mice there is an age-dependent influx of pro-inflammatory lymphocytes that can alter lacrimal gland and conjunctival structure and function, leading to decreased secretion and ocular surface disease [7,8,13]. In the present study, we demonstrated that similar changes in nerve structure and pro-inflammatory cytokine influx occur in the cornea, as in the lacrimal gland.

Tear secretion onto the ocular surface is regulated by a complex neural reflex that consists of the afferent sensory nerves of the cornea, trigeminal ganglion, trigeminal nucleus, superior salivatory nucleus, and the efferent parasympathetic and sympathetic nerves that innervate the lacrimal gland, conjunctival goblet, squamous epithelial cells, and corneal epithelial cells. Previously, we studied the efferent pathway in the absence of TSP-1 in mice, providing a model of autoimmune dry eye in which ocular surface homeostasis is disrupted [7]. Using this model, we found that TSP-1 is needed for the normal structure of the efferent parasympathetic and probably sympathetic nerves in the lacrimal gland as well as for function, as measured by depolarization-induced protein secretion. This dysfunction of lacrimal gland secretion contributes to the development of dry eye. In the present study, we focused on the afferent component of the neural reflex. Similar to the efferent nerves, we found that afferent nerve structure in the cornea is dependent upon TSP-1. We used two different methods, one in vivo confocal microscopy and one in vitro immunohistochemistry technique, that detect all the different types of nerves in the cornea. Both of these methods demonstrated fewer and discontinued nerve fibers in older TSP-1^−/−^ mice compared to WT control mice. Although we did not test function, because the structural changes in efferent lacrimal gland and afferent corneal nerves were similar, we propose that a reduction in the number and function of corneal nerves contributes to a suboptimal neural tear reflex in TSP-1^−/−^ mice. Thus, corneal afferent and lacrimal gland efferent nerves seem to be similarly affected in TSP-1^−/−^ mice.

Approximately half of primary afferent neurons in the cornea are reported to be peptidergic nociceptors containing SubP and/or CGRP [14,15,16], where the concentration and distribution of CGRP are reported higher than those of SubP [17,18,19]. Our data on these two neuropeptides showed a substantially decreased amount of CGRP in the corneas of older TSP-1^−/−^ as compared to WT mice of the same age. In contrast, no changes in SubP were found. Both SubP and CGRP are known to be involved in corneal wound healing, and delayed wound healing has been observed in TSP-1^−/−^ mice [20,21,22]. Importantly for the present study, CGRP is believed to play a key role in normal immunological functions by maintaining an anti-inflammatory state [23]. CGRP can function as an anti-inflammatory mediator that dampens excessive immune responses in septic shock, autoimmune diabetes, inflammatory bowel disease, and UV radiation-induced immunosuppression [24]. This neurotransmitter appears to work by preventing the development of T-cell driven autoimmune responses, as occurs in Sjögren’s Syndrome. Like in superior cervical ganglia neurons, CGRP works to increase TGF-β to produce regulatory T cells that dampen inflammation [25]. TSP-1 activates TGF-β to prevent inflammation in the ocular surface tissues. As CGRP has the same function as TGF-β, the decrease in corneal CGRP-containing nerves with disease progression in TSP-1^−/−^ mice is consistent with an increased immune response and could contribute to this increased response. It is not known, however, how the loss of TSP-1 decreases CGRP-containing nerves.

In contrast to CGRP, which is anti-inflammatory, SubP is pro-inflammatory. The blockage of SubP or its receptor in knockout mice or by pharmacological blockade has an anti-inflammatory effect in many chronic inflammatory diseases including arthritis, type 1 diabetes, inflammatory bowel disease, HSV-1-corneal infection, and corneal neovascularization [26]. SubP release stimulates Th1 and Th17 autoreactive T cells to produce IL17 and increases chemokines that recruit immune cells to the site of inflammation. In the present study, the level of SubP-containing neurons is unchanged with disease progression in TSP-1^−/−^ mice, allowing this neurotransmitter to continue its role in increasing inflammation. Our results suggest that CGRP- and SubP-containing nerves are differentially regulated, consistent with their distinct roles in the regulation of immune response.

It is possible, as in our study, to measure CGRP- and SubP-containing nerves separately in animal models. When studying corneal wound healing in mouse cornea, Cortina et al. found that after wounding, CGRP-containing nerves returned with PEDF + DHA treatment, but SubP-containing nerves did not [27]. This is further evidence that CGRP- and SubP-containing nerves are differentially regulated, consistent with their different functions.

Our results from the present study, combined with the different immunological roles of SubP and CGRP, suggest that it is critical to determine which type of neurotransmitter is altered when a decrease in corneal nerves is detected in various corneal pathologies or surgical treatments, including dry eye, corneal transplantation, and LASIK surgery. Although it is not possible to differentially evaluate SubP- and CGRP-containing nerves in humans, tear CGRP and SubP levels can be measured in tears. When CGRP and SubP were measured in tears after LASIK, CGRP levels increased whereas SubP levels were unchanged [28]. These findings could explain why the ocular surface remains relatively quiet after this type of surgery. This result is consistent with our current study in that CGRP- and SubP-containing nerves are differentially regulated.

In the present study we found that the structure of corneal afferent nerves was disrupted in the absence of TSP-1, suggesting that TSP-1 is needed as an extracellular matrix protein either for appropriate peripheral nerve development or for nerve repair. There is little published work on the effect of TSP-1 on peripheral nerves, except our previous study on lacrimal gland efferent parasympathetic nerves in which TSP-1 was also needed for nerve growth or repair [8]. Several publications have found, however, that TSP-1 is needed for synapse formation in the central nervous system, with TSP-1 being secreted by astrocytes [29,30,31]. The secreted TSP-1 interacts with the neuronal calcium channel subunit *α*26-1 that may act as the on switch for synapse formation. The synapses for afferent corneal nerves project to the trigeminal ganglion. As trigeminal ganglion neurons do not have synapses, TSP-1 must work by a different mechanism to affect corneal efferent nerves. TSP-1 does have multiple binding partners in addition to *α*26-1, including ApoER2, CD36, CD47, heparin, latent TGF-β, LRP1/CRT, neuroligin, notch, and VLDR [29], that could function in the regulation of corneal afferent nerve growth. More research is needed to determine the mechanism by which TSP-1 affects corneal sensory nerves, including the cellular source of TSP-1, the neural binding partner, and the cellular location (trigeminal ganglion, nerve axon, nerve endings, or supporting cells) of the interaction.

In addition to TSP-1 directly affecting corneal afferent nerve growth or repair by a TSP-1 protein interaction, TSP-1 could alter these nerves indirectly by its effect on inflammation. In the conjunctiva, in the absence of TSP-1, there is an increase in inflammatory cell infiltration and in pro-inflammatory cytokine production from the lack of activation of TGF-βII (the main TGF-β isoform in the ocular surface) [13,32]. In the cornea, the expression of three different pro-inflammatory cytokines—TNF-α, MCP-1, and MIP-2—was examined and only MIP-2 was elevated in young TSP-1 compared to WT cornea; however, all three cytokines were increased in older mice. Furthermore, CD11b+ cells were not altered in older TSP-1^−/−^ mouse corneas. These results argue against an indirect effect of TSP-1 on nerves via the activation of MCP and TNF-α-dependent pro-inflammatory pathways in young mice. TSP-1 could play an effect in addition to the direct effect of TSP-1 binding for MIP-2-dependent action in young mice and for all three cytokines measured in older mice. More research, however, on different time points, cytokines, and immune cells is needed to determine if the activation of inflammation plays a role in the damage to corneal nerve growth and repair.

In conclusion, we found that TSP-1 is needed for the development or repair of corneal afferent nerves, especially in the population of nerves containing CGRP as a neurotransmitter. The changes in these nerves occur in older TSP-1^−/−^ mice as the corneal expression of pro-inflammatory cytokines increases, but there is no indication of inflammatory cell activation. TSP-1 could control corneal CGRP-containing nerve development and repair by a direct effect as a matricellular protein interacting with its binding partners on the nerves and/or indirectly by preventing the development of an inflammatory environment.

## 4. Materials and Methods

### 4.1. Animals

Female C57BL/6 and TSP-1^−/−^ mice (C57BL/6 background) aged 4–7 weeks (young, pre disease onset) and 9–12 weeks (older, post disease onset) were used in this study. C57BL/6 mice were purchased from Jackson Laboratories (Bar Harbor, ME, USA). TSP-1^−/−^ mice used were originally developed by Dr. Jack Lawler (Harvard Medical School, Boston, MA, USA) and were bred in-house in a pathogen-free facility at Schepens Eye Research Institute, Boston, MA or were obtained from Dr. Sharmila Masli (Boston University, Boston, MA, USA). All experiments conformed to the National Institutes of Health guide for the care and use of laboratory animals (NIH Publications No. 8023, revised 1978) and were approved by the Schepens Eye Research Institute Animal Care and Use Committee, project idenfitication code S472-1219, approval date 28 March 2017.

### 4.2. Hemotoxylin and Eosin Staining

Whole eyes were excised and fixed in 4% paraformaldehyde at room temperature for 40 min. Fixed eyes were washed in phosphate buffered saline (PBS) and cryoprotected with sucrose. The tissue was embedded in optimal cutting temperature (OCT) media (Tissue-Tek, Torrance, CA, USA), and snap-frozen as previously described. Six to eight micron corneal sections were cut and stained with hematoxylin and eosin reagent. The slides were viewed by light microscopy (Eclipse E80i; Nikon, Tokyo, Japan).

### 4.3. Quantitative Real-Time PCR

Three corneas were harvested from each group: 4-week-old (young, before disease onset), 12-week-old (older, after disease onset) TSP-1^−/−^ mice, (C57BL/6 background) and C57BL/6 mice. Total RNA was isolated from the corneas using a commercially available kit (KAPA SYBR^®^ FAST qPCR Kit (KK4602)) and reverse transcribed using a Super Script^®^ III Kit (Invitrogen Life Technologies, Carlsbad, CA, USA). Real-time PCR was performed to determine the relative quantitative expression of dry eye-associated inflammatory molecules (TNF-α, MCP-1, and MIP-2) by using Universal PCR Mastermix (Invitrogen, Carlsbad, CA, USA) and predesigned primers on an Ependorf Mastercycler (Ependorf North America, New York, NY, USA). Data were analyzed by a comparative threshold cycle method and normalized by GAPDH as an internal control.

### 4.4. In Vivo Confocal Microscopy

For in vivo confocal microscopy, mice were anesthetized via an intraperitoneal injection of ketamine (120 mg/kg; Phoenix Scientific, St. Joseph, MO, USA) and xylazine (20 mg/kg; Phoenix Scientific). Eyes were treated with a drop of Proparacaine 0.5% Ophthalmic Solution, a topical anesthetic. A drop of Genteal (Novartis, St. Louis, MO, USA) lubrican puralube eye gel was placed on the tip of the objective lens to maintain immersion contact between the objective lens and the eye. The contralateral eye was moisturized with the same gel and subsequently imaged. During the procedure, anesthetized mice were placed on a heated platform to maintain physiological temperature. Images were acquired using an HRT II/RCM (Heidelberg Engineering GmbH, Heidelberg, Germany) in vivo confocal microscope with a diode laser with a wavelength of 670 nm and a 60× objective immersion lens. An area of 400 × 400 μm with a transverse optical resolution of approximately 1 mm/pixel was imaged. For all eyes, 30 images of each layer of the cornea (total 150 images), including the superficial and basal epithelium, anterior and posterior stroma, and endothelium, were recorded.

### 4.5. In Vitro Immunohistochemical Analysis

To analyze corneal nerve structure and number, at least three corneas were collected from each group: young (aged between 4 and 7 weeks) WT and young TSP-1^−/−^, older (aged between 9 and 12 weeks) WT and older TSP-1^−/−^ mice. Corneas were dissected and fixed in 4% paraformaldehyde for 40 min at room temperature. Four radial relaxing incisions were made before the corneas were washed with PBS with 0.1% bovine serum albumin (BSA), and then blocked for 2 h with 1% BSA in PBS and 0.1% Triton X-100. The corneas were then incubated with either anti-beta-tubulin Class III (Millipore/Chemicon rabbit anti-beta III Tubulin or T2200 Sigma, St. Louis, MO, USA) at a dilution of 1:200, anti-CGRP (Goat polyclonal Cat# 1720-9007 BioRad, Hercules, CA, USA) or anti-SubP (rat monoclonal Cat# MAB356 EMD Millipore, Burlington, MA, USA) at a dilution of 1:200 primary antibody for 72 h at 4 °C.

After four washes in PBS containing 0.1% BSA, corneas were either incubated with the appropriate secondary antibody: DyLight 488 (Donkey Anti-Rabbit IgG, Cat# 711-485-152, Jackson Immuno Research, West Grove, PA, USA) at a dilution of 1:100 or DyLight 594 (Goat Anti-Rabbit IgG, Jackson Immuno Research, Cat# 111-515-003) at a dilution of 1:200 overnight at 4 °C. The corneas stained with anti-SubP antibody were incubated with secondary antibody Cy2 (Goat Anti-Rat, Cat# ab6952, Abcam, Cambridge, UK) at a dilution of 1:200, and those stained with anti-CGRP antibody were incubated with secondary antibody Cy3 (Anti-Goat) at a dilution of 1:400. The corneas were then rinsed in PBS and mounted in a medium containing DAPI (VectaShield; Vector Laboratories, Burlingame, CA, USA).

Corneas were viewed by confocal imaging (TCS-SP2, Leica Microsystems, Bannockburn, IL, USA). Four fields of view at the level of the subbasal nerve plexus near the periphery of the cornea were photographed (2007 IOVS YuCQ Transgenic corneal neurofluorescence in mice). To view CGRP and SubP, images were acquired at 200× magnification. The density of corneal nerves was measured using NIH ImageJ with the NeuronJ plugin using established methods [10]. Two different methods were used, one for analyzing studies using anti-beta III Tubulin only and another when analyzing CGRP and SubP in the same study. The first method involved the quantification of neural density by tracing nerves according to established methods based on the tubular nature of the staining with anti-Class III β-tubulin [10]; the results are reported as average mm nerve/mm^2^ ± SD. The second method involved the calculation of the average percent of area staining for the specific neurotransmitter using ImageJ (Fiji, https://imagej.nih.gov/ij/), due to the discrete nature of neurotransmitter-containing vesicles stained by anti-CGRP or anti-SubP antibodies.

### 4.6. Anti-CD11b Antibody Analysis

To analyze the monocyte number of corneas, one eye from three mice per group were excised and fixed in acetone. Corneas were blocked in 2% bovine serum albumin for 45 min and then incubated with fluorescein isothiocyanate (FITC)-conjugated rat anti–mouse CD11b (marks monocyte derived cells, BD Pharmingen, San Diego, CA, USA) at ratio of 1:100 overnight at 4° [33]. Corneas were washed and mounted using a medium containing DAPI to indicate cell nuclei. The slides were viewed by a fluorescence microscope (Eclipse E80i; Nikon, Tokyo, Japan) at a magnification of 40×. CD11b+ cells were counted in eight areas in the periphery (0.5-µm area from the limbus) and two areas in the center (central 2-µm area) of each cornea in a masked fashion [33]. The mean number of cells was obtained by averaging the total number of cells in all the areas studied, and the result was expressed as the number of positive cells per square millimeter.

### 4.7. Statistical Analyses

CD 11b+ cells/mm^2^ were measured and the results were analyzed by unpaired *t*-test. A *p*-value < 0.05 was deemed statistically significant. Results for in vitro immunohistochemical analysis of corneas were analyzed by one-way analysis of variance (ANOVA), as appropriate, followed by the Scheffe post-hoc test. A *p*-value < 0.005 was deemed statistically significant. Results of qRT-PCR were analyzed by one-way analysis of variance (ANOVA), as appropriate, followed by the Scheffe post-hoc test. A *p*-value < 0.05 was deemed statistically significant.

## Supplementary Materials

Supplementary materials can be found at http://www.mdpi.com/1422-0067/19/10/3191/s1.
